# Molecular identification and characterization of *Sarcocystis* spp. in horsemeat and beef marketed in Japan

**DOI:** 10.1051/parasite/2018026

**Published:** 2018-05-08

**Authors:** Rie Murata, Jun Suzuki, Ayako Hyuga, Takayuki Shinkai, Kenji Sadamasu

**Affiliations:** Department of Microbiology, Tokyo Metropolitan Institute of Public Health, 3-24-1 Hyakunincho, Shinjuku-ku, Tokyo 169-0073 Japan

**Keywords:** *Sarcocystis fayeri*, *Sarcocystis cruzi*, 18S rRNA, mtDNA *cox1*, intraspecific variation

## Abstract

Human *Sarcocystis* infections are known to be caused by the ingestion of raw or undercooked beef or pork containing mature sarcocysts of *Sarcocystis hominis* or *S. suihominis*, respectively. In addition, several cases of parasitic food poisoning in Japan have recently been reported after consumption of raw horsemeat containing sarcocysts of *S. fayeri*. In this study, the presence of sarcocysts in 28 horsemeat and 121 beef samples collected in Tokyo was investigated. Sarcocysts of *S. fayeri* were found in 16 horsemeat samples. Sarcocysts of *S. hominis* were not detected in beef samples, while sarcocysts of *S. cruzi* were detected in 60 beef samples. In addition, *S. hirsuta* and *S. bovini* were isolated only from New Zealand beef samples. Bradyzoites in sarcocysts collected from 62/73 sarcocyst-positive refrigerated horsemeat and beef samples were determined to be viable. Molecular analysis of *S. fayeri* 18S rRNA gene sequences revealed that intraspecific variation among eight individual bradyzoites from a single sarcocyst was as high as 9.8%. In contrast, mitochondrial cytochrome c oxidase subunit 1 (mtDNA *cox1*) gene sequences from the six fragments of a single sarcocyst were 100% identical. Sarcocysts of *S. bovini* isolated from beef also exhibited intraspecific variation in 18S rRNA gene sequences and had to be cloned before sequencing, while mtDNA *cox1* gene sequences were obtained by direct sequencing. Therefore, we conclude that molecular analysis of the mtDNA *cox1* gene is the most useful for identification of *Sarcocystis* species. This study provides the first published partial sequence of the *S. fayeri* mtDNA *cox1* gene.

## Abstract (fr)

Les infections humaines par *Sarcocystis* sont causées par l’ingestion de viande de bœuf ou de porc, crue ou insuffisamment cuite, contenant des sarcocystes mûrs de *Sarcocystis hominis* ou de *S. suihominis*, respectivement. En outre, plusieurs cas d’intoxication alimentaire parasitaire ont récemment été signalés au Japon après consommation de viande de cheval crue contenant des sarcocystes de *S. fayeri*. Dans cette étude, la présence de sarcocystes dans 28 échantillons de cheval et 121 échantillons de bœuf collectés à Tokyo a été étudiée. Des sarcocystes de *S. fayeri* ont été trouvés dans 16 échantillons de viande de cheval. Des sarcocystes de *S. hominis* n’ont pas été détectés dans les échantillons de bœuf, alors que des sarcocystes de *S. cruzi* ont été détectés dans 60 échantillons de bœuf. De plus, *S. hirsuta* et *S. bovini* n’ont été isolés qu’à partir d’échantillons de bœuf néo-zélandais. Les bradyzoïtes des sarcocystes prélevés sur 62/73 échantillons de viande de cheval et de viande de bœuf réfrigérés positifs aux sarcocystes ont été jugés viables. L’analyse moléculaire des séquences du gène de l’ARNr de *S. fayeri* a révélé que la variation intraspécifique entre huit bradyzoïtes individuels d’un seul sarcocyste atteignait 9,8 %. En revanche, les séquences du gène de la sous-unité 1 de la cytochrome c oxydase mitochondriale (ADNmt *cox1*) provenant des six fragments d’un seul sarcocyste étaient identiques à 100 %. Les sarcocystes de *S. bovini* isolés du bœuf présentaient également une variation intraspécifique des séquences du gène de l’ARNr 18S et ont dû être clonés avant le séquençage, tandis que les séquences du gène *cox1* ont été obtenues par séquençage direct. Par conséquent, nous concluons que l’analyse moléculaire du gène *cox1* est la plus utile pour l’identification des espèces de *Sarcocystis*. Cette étude fournit la première séquence partielle publiée du gène *cox1* de *S. fayeri*.

## Introduction

The genus *Sarcocystis* includes intracellular protozoan parasites that parasitize many different animals, including mammals, birds, and fish. They have an obligatory prey-predator (intermediate-definitive) two-host life cycle; they reproduce asexually in herbivores and omnivores as intermediate hosts, and sexually in carnivores as definitive hosts [2,4]. Identification of *Sarcocystis* spp. is based on morphological characteristics of the sarcocyst and host information, such as the host species and the prepatent period [2]. However, morphological diagnosis of sarcocysts is difficult as some *Sarcocystis* spp. share sarcocyst morphologies and intermediate hosts; for example, *S. hominis*, *S. bovifelis,* and *S. bovini* are all found in cattle. Additionally, morphological changes occur with sarcocyst aging and in relation to the locations of intermediate hosts [2,4]. Recently, molecular methods, particularly sequencing of 18S ribosomal RNA (rRNA), have been increasingly used to complement morphological data in the identification of *Sarcocystis* species [6].

Sarcocystosis in humans presents as two forms. The first is intestinal sarcocystosis caused by *Sarcocystis hominis* and *Sarcocystis suihominis*, which use humans as definitive hosts. The other is muscular sarcocystosis caused when humans become dead-end hosts for non-human *Sarcocystis* spp. after the accidental ingestion of oocysts. Recently, food poisoning manifesting as transient vomiting and diarrhea has been reported after consumption of raw horsemeat in Japan [8]. *Sarcocystis fayeri* sarcocysts in horsemeat were found to induce diarrhea following oral inoculation of bradyzoites in rabbits [15]. In this study, we investigated the presence of *S. fayeri* sarcocysts in horsemeat (*Equus caballus*) and of *S. hominis* sarcocysts in beef (*Bos taurus*). Additionally, sarcocysts were identified and characterized by molecular methods.

## Materials and methods

### Meat samples

A total of 28 horsemeat samples were obtained from wholesale merchants, retail meat shops, and mail-order vendors in Japan from June to December 2012. Among these, 13 were refrigerated Japanese samples, 13 were imported from Canada (six refrigerated samples and seven frozen samples), and two were refrigerated samples imported from Italy (Table 1). A total of 121 refrigerated beef samples were obtained from retail meat shops and mail-order vendors in Japan from May 2013 to December 2015. These consisted of 75 Japanese samples (28 hearts, ten tongues, 12 diaphragms, and 25 rounds), 21 imported samples from the United States of America (USA) (ten tongues, nine diaphragms, and two rounds), 20 imported samples from Australia (four tongues, four diaphragms, ten rounds, and two rib roasts), and five imported samples from New Zealand (five rounds) (Table 2).

**Table 1:** Detection of *Sarcocystis fayeri* from horsemeat.

Country of origin	refrigerated / frozen	Tested	No. of positive samples	No. of viable bradyzoite samples
Japan	refrigerated	13	4	3
Canada	refrigerated	6	6	5
	frozen	7	5	0
Italy	refrigerated	2	1	1

**Table 2:** Detection of *Sarcocystis* spp. from refrigerated beef.

	Muscle part	Tested	No. of positive samples (%)						
				
Country of origin			*S. cruzi*		*S. hirsuta*		*S. bovini*		No. of viable bradyzoite samples
Japan	heart	28	22	(78.6)	0		0		22
	tongue	10	6	(60.0)	0		0		2
	diaphragm	12	4	(33.3)	0		0		4
	round	25	2	(8.0)	0		0		1
	total	75	34	(45.3)	0		0		29
United States of America	tongue	10	9	(90.0)	0		0		8
	diaphragm	9	7	(77.8)	0		0		7
	round	2	0		0		0		0
	total	21	16	(76.2)	0		0		15
Australia	tongue	4	3	(75.0)	0		0		3
	diaphragm	4	2	(50.0)	0		0		1
	round	10	3	(30.0)	0		0		1
	rib roast	2	1	(50.0)	0		0		1
	total	20	9	(45.0)	0		0		6
New Zealand	round	5	1^a^	(20.0)	1	(20.0)	3	(60.0)	3

a *S. cruzi*, *S. hirsuta*, and *S. bovini* were detected in this sample.

### Isolation and microscopic examination of sarcocysts

From each horsemeat and beef sample, 5 slices (app. 5 × 3 × 1 cm) were examined under a stereoscopic microscope. Sarcocysts were isolated with small tweezers and transferred to a drop of phosphate-buffered saline (PBS) on a glass slide. Sarcocysts were determined to be thin- or thick-walled under a light microscope without a coverslip.

The viability of bradyzoites in sarcocysts was assayed using two methods. In the trypan blue dye exclusion assay, a bradyzoite suspension on a glass slide was added to an equal volume of 0.4% trypan blue solution (Thermo Fisher Scientific, USA), and immediately examined under a light microscope. In the second assay, the resistance of bradyzoites in sarcocysts to enzymatic digestion was determined. The sarcocyst was suspended in 1 mL of digestive fluid (1 g of pepsin, 0.8 mL of concentrated HCl, and PBS to 50 mL), incubated at 37 °C for 30 min, and centrifuged at 9,000 g for 1 min before the supernatant was discarded. The pellet was immediately observed under a light microscope to determine whether bradyzoites were digested.

### DNA extraction

For DNA extraction from bradyzoites isolated from horsemeat, one sarcocyst was divided into six parts with a disposable scalpel. Three of the six cyst fragments were individually placed into 0.8% NaCl in a Petri dish, and each cyst fragment was broken apart using small tweezers in order to release the bradyzoites. Bradyzoites in suspension were counted with a Neubauer improved cell counting chamber and adjusted to a final concentration of 1–3 bradyzoites in 5 µL of 0.8% NaCl. The adjusted bradyzoite suspension (5 µL) was placed in a 0.2 mL tube. Next, 25 µL of a DNA extraction mixture containing 3 µL of 10 × Ex Taq Buffer (Takara Bio, Japan), 5 µL of 10% Triton X-100, and 17 µL of H_2_O was added and incubated at 95 °C for 30 min with a heating block.

DNA was extracted from individual sarcocysts and from each of the six individual cyst fragments (including the three from which bradyzoite DNA had been extracted) using the QIAamp DNA Mini Kit (Qiagen, Netherlands).

### PCR amplification

For the first PCR amplification of 18S rRNA from *S. fayeri* bradyzoites (app. 1900 bp), DNA templates were each added to a 20 µL reaction mixture containing 2 µL of 10 × Ex Taq Buffer, 4 µL of 2.5 mM each dNTP, 2 µL of 10 µM each primer (ER1B1 and PrimerB; Table 3), 0.2 µL of 5 U/µL Ex Taq (Takara Bio, Japan), and 9.8 µL H_2_O. The second PCR was performed in a 50 µL reaction mixture containing 1 µL of the first PCR product, 1 U of Ex Taq, 0.4 µM of each primer, and 0.2 mM of each dNTP. Primer pairs included ER1B1/Sar18S619R (app. 600 bp), 18S1F/18S11R (app. 950 bp), Sar-UF/18S11R (app. 950 bp), Sar-UF/ShR2 (app. 850 bp), Sar18S674F/Primer 1H (app. 650 bp), and Sar18S1149F/PrimerB (app. 700 bp) (Table 3). The following cycling parameters were used: initial denaturation at 94 °C for 5 min; 35 cycles of 94 °C for 30 s, 60 °C for 30 s, and 72 °C for 1 min; and a final extension at 72 °C for 5 min.

**Table 3:** Oligonucleotide primers used for PCR.

DNA region	Primer name	Orientation	Primer sequence (5’ to 3’)	Reference
18S rRNA	ERlB1	Forward	ACCTGGTTGATCCTGCCAG	[10]
	PrimerB	Reverse	GATCCTTCTGCAGGTTCACCTAC	[10]
	18S1F	Forward	GGATAACCGTGGTAATTCTATG	[13]
	18S11R	Reverse	TCCTATGTCTGGACCTGGTGAG	[13]
	Primer 3L	Forward	CTAGTGATTGGAATGATGGG	[18]
	Primer 1H	Reverse	TATCCCCATCACGATGCATAC	[18]
	NSF1179/18	Forward	AATTTGACTCAACACGGG	[9]
	Sar-UF	Forward	GCTTTCGACGGTAGTGTATTGGA	This study
	Sar18S674F	Forward	GCGAAAGCATTTGCCAARGATG	This study
	Sar18S1149F	Forward	AGTATGGTCGCAAGGCTG	This study
	Sar18S619R	Reverse	ACGCTATTGGAGCTGGAATTAC	This study
	ShR2	Reverse	AGTTTCAGCCTTGCGACCATA	This study
	18SR11-1	Reverse	TCCCATGTCTGGACCTGGTGAG	This study^a^
mtDNA *cox1*	SF1	Forward	ATGGCGTACAACAATCATAAAGAA	[6]
	SR5	Reverse	TAGGTATCATGTAACGCAATATCCAT	[6]
	COIRm	Reverse	CCCAGAGATAATACAAAATGGAA	[6]
	CoxS1R	Reverse	TTACCCATGACCACACCTGTAGTACC	[6]
	Sarf-COX1F	Forward	TTCTCTACGTCTGGTCGATAGT	This study
	Sarf-COX1R	Reverse	AATACTATCGACCAGACGTAGAGA	This study
	Sarc-COX1R	Reverse	AGTATGAGCATTAAAGCCGTGAA	This study
	Sarh-COX1R	Reverse	AACATACAGCACTCCAGATT	This study
	Sarb-COX1R	Reverse	AGACGTACAGAATACCACATC	This study

a Modification of primer 18S11R described in reference [13].

PCR of sarcocysts and cyst fragments was used to amplify the 18S rRNA gene and the mitochondrial cytochrome c oxidase subunit 1 (mtDNA *cox1*) gene. Amplification was performed with the same method as that used for the second PCR of bradyzoites. The 18S rRNA gene was amplified using the primer pair ER1B1/PrimerB (Table 3). The cycling parameters were the same as those for bradyzoites. The amplified *Sarcocystis* 18S rRNA gene was cloned using the Qiagen PCR Cloning Kit (Qiagen, Netherlands). The mtDNA *cox1* gene was amplified using the forward primer SF1 and reverse primers SR5 (for *S. fayeri*), COIRm (for *Sarcocystis* *cruzi* and *Sarcocystis* *bovini*), and CoxS1R (for *Sarcocystis hirsuta*) as previously described (Table 3) [6]. The following cycling parameters were used: initial denaturation at 95 °C for 15 min; 45 cycles of 94 °C for 30 s, 52 °C for 30 s, and 72 °C for 90 s; and a final extension at 72 °C for 10 min.

### Sequence and phylogenetic analysis

PCR products of the 18S rRNA and mtDNA *cox1* genes were sequenced using the ABI Prism BigDye Terminator v3.1 Cycle Sequencing Ready Reaction Kit and ABI Prism 3500 Genetic Analyzer (Thermo Fisher Scientific, USA). Primers used for sequence analysis included Primer 3L, NSF1179/18, 18SR11-1, Sarf-COX1F, Sarf-COX1R, Sarc-COX1R, Sarh-COX1R, and Sarb-COX1R, in addition to PCR primers (Table 3).

For analysis of 18S rRNA sequences, bases corresponding to positions 153–1283 of the *S. cruzi* 18S rRNA gene (KT901167) were used. For analysis of mtDNA *cox1*, sequences longer than 812 bp were truncated at their 3′ end. Multiple alignment of the 18S rRNA sequences of the *Sarcocystis* species was conducted as previously reported with ClustalW, using a gap opening penalty of 10 and a gap extension penalty of 0.2 for both pairwise and multiple alignment [7,17]. The phylogenetic analyses of the 18S rRNA and mtDNA *cox1* genes were performed using the maximum likelihood (ML) and maximum parsimony (MP) methods within MEGA version 6 [16]. As the ML analytical model gave the highest log likelihood, the ML tree was derived using a general time-reversible model employing estimates of the proportion of invariable sites and a Gamma distribution with five rate categories; statistical support was evaluated using bootstrapping with 1,000 replicates. The phylogeny based on MP analysis was tested with 1,000 replicates and the Tree-Bisection-Regrafting algorithm.

**Figure 1:**
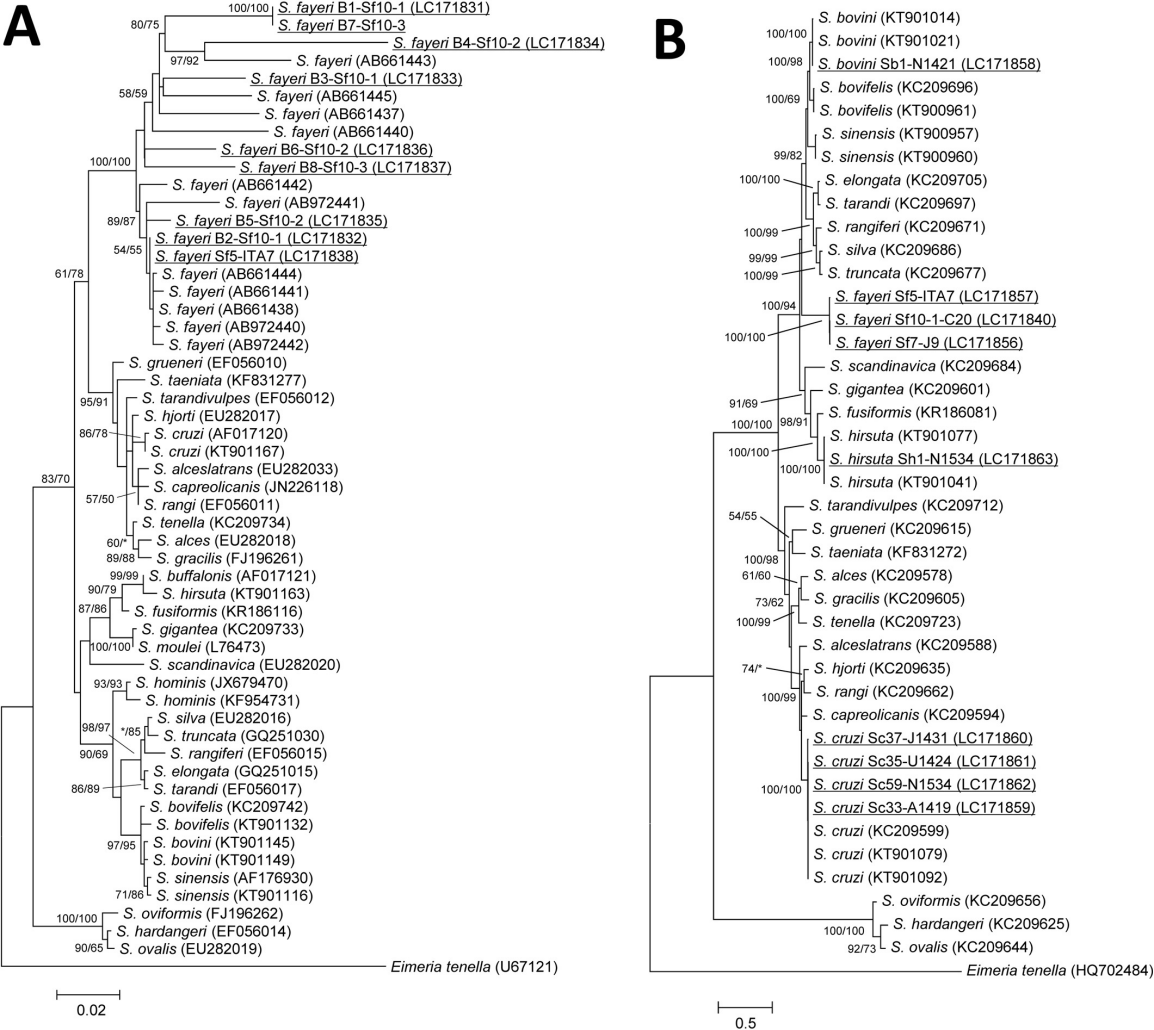
Phylogenetic relationships obtained by maximum likelihood (ML) analysis of *Sarcocystis* 18S rRNA (A) and mtDNA *cox1* (B) gene sequences. ML trees were derived using a general time-reversible model employing estimates of the proportion of invariable sites and the Gamma distribution. Significant bootstrap support (> 50%) from 1,000 replicates of the ML and the maximum parsimony (MP) method are shown above each node in the order ML/MP. Support for a node of less than 50% is indicated by an asterisk. Underlined samples are those sequenced in this study. The scale bar represents the distance in substitutions per nucleotide. GenBank accession numbers are shown in parentheses.

GenBank accession numbers of the *Sarcocystis* species used in the phylogenetic analysis are shown in Figure 1. *Eimeria tenella* sequences (GenBank accession numbers U67121 and HQ702484 for the 18S rRNA and mtDNA *cox1* genes, respectively) were used as outgroups to root the final phylogenetic tree.

## Results

### Microscopic detection of *Sarcocystis* in horsemeat and beef

Sarcocysts isolated from horsemeat exhibited thick and radially striated cyst walls, consistent with *S. fayeri* sarcocysts [2,3]. These sarcocysts were found in 16 samples, including four Japanese, 11 Canadian, and one Italian sample. Of these, bradyzoites in sarcocysts collected from three refrigerated Japanese, five refrigerated Canadian, and one refrigerated Italian sample were determined to be viable, while bradyzoites collected from one refrigerated Japanese, one refrigerated Canadian, and five frozen Canadian horsemeat samples were dead (Table 1).

**Figure 2:**
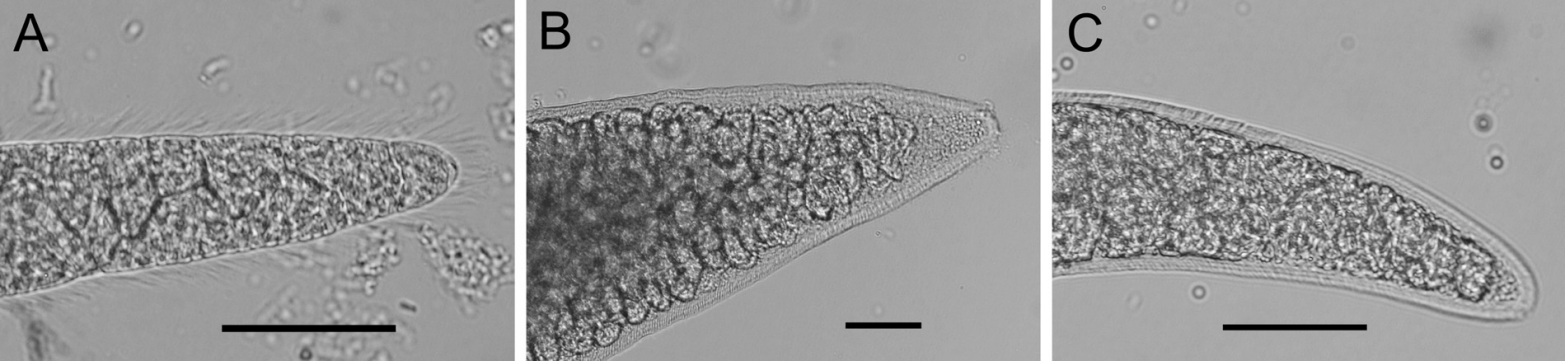
Morphology of *Sarcocystis* spp. isolated from beef. A, *Sarcocystis cruzi*; B, *Sarcocystis hirsuta*; C, *Sarcocystis bovini*. Bar: 50 µm.

Sarcocysts isolated from beef could be morphologically classified into three types [2,5,12]. The first type exhibited thin cyst walls with hair-like protrusions, consistent with *S. cruzi* sarcocysts (Figure 2A). These sarcocysts were found in 34 Japanese (22 hearts, six tongues, four diaphragms, and two rounds), 16 US samples (nine tongues and seven diaphragms), nine Australian samples (three tongues, two diaphragms, three rounds, and one rib roast), and one New Zealand sample (round). The second type of cysts was macroscopically visible with thick and radially striated cyst walls, consistent with *S. hirsuta* sarcocysts (Figure 2B). These sarcocysts were found in a single New Zealand beef sample that contained all three types of sarcocysts. The third type exhibited thick and radially striated cyst walls, similar to *S*. *hominis* sarcocysts (including *Sarcocystis bovifelis* and *S. bovini*) (Figure 2C). This type was found in three New Zealand beef samples. Non-viable bradyzoites in sarcocysts were found in five Japanese samples (four tongues and one round), one US sample (tongue), and three Australian samples (one diaphragm and two rounds), while the bradyzoites collected from the remaining beef samples were determined to be viable (Table 2).

### Molecular identification of sarcocysts

Of the 16 sarcocyst-positive horsemeat samples, we were unable to conduct molecular analysis on the sarcocysts from two Japanese samples. Five sarcocysts (Sf10-C20 to Sf14-C20) were isolated from a single Canadian horsemeat sample (C20), and one of these was divided into six parts (Sf10-1-C20 to Sf10-6-C20) before DNA was extracted from each of the six individual cyst fragments. From the other 13 horsemeat samples, individual, intact sarcocysts were used for molecular analysis. Of the 23 cyst or cyst-fragment DNA samples, a clear, single-peak chromatogram of a 1861 bp sequence of the 18S rRNA gene was obtained from only a single sarcocyst (Sf5-ITA7), isolated from an Italian horsemeat sample. PCR amplification of the other sarcocyst DNA samples isolated from Canadian and Japanese horsemeat yielded ambiguous sequences, including multiple peaks at several positions in the chromatograms. Amplicons from each of these sarcocysts were cloned, and five clones derived from one sarcocyst isolated from Canada and 12 clones from one sarcocyst isolated from Japan were sequenced. In the 18S rRNA phylogenetic analyses, these sequences clustered with the *S. fayeri* group, and genetic distances among clones from Canada and Japan, generated with the Kimura 2-parameter model, were 0.003–0.069 and 0.006–0.108, respectively.

Eight sequences with clear, individual chromatogram peaks were obtained by sequencing of individual bradyzoites (B1-Sf10-1–B3-Sf10-1, B4-Sf10-2–B6-Sf10-2, B7-Sf10-3, and B8-Sf10-3) released from a single sarcocyst (Sf10) using nested PCR of the 18S rRNA gene. Of these, two sequences (B1-Sf10-1 and B7-Sf10-3) were 100% identical. The other six sequences exhibited high intraspecific variation when compared with each other. Among the pairwise genetic distances between the eight sequences, the highest genetic distance between a pair of sequences was 0.094, and the average genetic distance was 0.059. In the phylogenetic tree of 18S rRNA gene sequences, these eight sequences formed several clusters with reference strains of *S. fayeri* (Figure 1).

Sequence analysis was also performed on the mtDNA *cox1* gene from the 23 cysts or cyst fragments isolated from horsemeat samples. These 23 cyst samples were identified as *S. fayeri* based on similarity searches of the 18S rRNA gene sequences using BLAST. The sequences from the six cyst fragments (Sf10-1-C20 to Sf10-6-C20) of a single sarcocyst (Sf10) from one Canadian horsemeat sample (C20) were 100% identical. In contrast, sequences from four sarcocysts (Sf11-C20 to Sf14-C20) isolated from the same sample (C20) and from 13 sarcocysts isolated from the other horsemeat samples contained 5–8 and 2–9 base substitutions, respectively, when compared with the sequence obtained from all six cyst fragments (Table 4). In the mtDNA *cox1* phylogenetic tree, the 23 cyst and cyst-fragment sequences formed a monophyletic cluster (Figure 1B; only three sarcocyst samples are shown). Eight partial sequences of the 18S rRNA gene and 18 partial sequences of the mtDNA *cox1* gene of *S. fayeri* were deposited in GenBank with accession numbers LC171831–LC171838 and LC171840–LC171857, respectively.

**Table 4:**
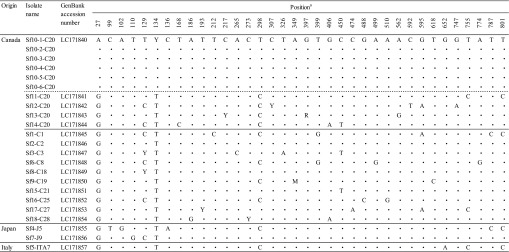
Summary of polymorphic sites in the mtDNA *cox1* gene (812 bp) of *Sarcocystis fayeri* sarcocysts isolated from horsemeat.

Dots indicate matches to the Sf10-1-C20 sequence. ^a^ Nucleotide position from the 5’ end of LC171840.

On the basis of the molecular analysis of *S. fayeri* sarcocysts, the mtDNA *cox1* gene was more useful in the identification of sarcocysts than the 18S rRNA gene. Therefore, sarcocysts isolated from beef were analyzed using sequences of the mtDNA *cox1* gene. In the phylogenetic tree of the mtDNA *cox1* gene, sarcocysts exhibiting the morphological characteristics of the *S. cruzi*, *S. hirsuta*, and *S. hominis* types clustered with *S. cruzi*, *S. hirsuta*, and *S. bovini*, respectively (Figure 1B; only four *S. cruzi*, one *S. hirsuta*, and one *S. bovini* isolates are shown). However, there were no mtDNA *cox1* gene sequences for *S. hominis* found in GenBank. Therefore, *S. hominis*-type sarcocysts were further analyzed by sequencing the 18S rRNA gene, resulting in ambiguous sequences, which were further delineated by cloning. Five clones of one *S. homini*s-type sarcocyst were sequenced, and the genetic distances among them were 0.005–0.016. In the 18S rRNA phylogenetic analyses, these five sequences belonged to the same cluster of *S. bovini,* and this cluster was separate from the *S. hominis* sequences. Partial mtDNA *cox1* gene sequences of four *S. cruzi*, one *S. hirsuta*, and one *S. bovini* isolate were deposited in GenBank with accession numbers LC171859–LC171862, LC171863, and LC171858, respectively. Partial 18S rRNA gene sequences of four *S. cruzi* and one *S. hirsuta* isolate were deposited in GenBank with accession numbers LC171827–LC171830 and LC171839, respectively.

## Discussion

### Detection of *Sarcocystis* spp. in horsemeat and beef

In this study, although it was difficult to obtain a large sample size, *S. fayeri* sarcocysts were found more often in Canadian horsemeat than in Japanese horsemeat, consistent with the results of previous research, in which the prevalence of *S. fayeri* sarcocyst infections in Japanese horses was 42.9% and that in horses that were born abroad and bred in Japan was 100% [14]. Bradyzoites in sarcocysts collected from 9/11 refrigerated horsemeat samples infected with *S. fayeri* were viable. Thus, vomiting and diarrhea may be induced following the ingestion of raw refrigerated horsemeat.

Among beef samples, *S. hominis* sarcocysts were not detected, and *S. cruzi* was the most prevalent species. The prevalence of *S. cruzi* sarcocyst infections in cattle were reported to be significantly higher in hearts than those in other organs [11]. In this study, the prevalence of *S. cruzi* sarcocyst infections was higher in Japanese beef hearts than those in tongues, diaphragms, and rounds. Comparison of Japanese and imported beef samples revealed that the prevalence of *S. cruzi* sarcocyst infections was particularly high in US tongues and diaphragms and in Australian tongues. Bradyzoites in sarcocysts collected from most refrigerated beef samples infected with *S. cruzi* were determined to be viable. In addition to *S. cruzi*, *S. hirsuta* and *S. bovini* were isolated from New Zealand beef samples, and these bradyzoites were viable. Sarcocysts of *S. bovini*, *S. hominis*, and *S. bovifelis* in cattle have thick and radially striated cyst walls. The morphological differences among these sarcocysts are difficult to distinguish by light microscopy. Thus, molecular analysis is necessary for the identification of *S. hominis*, which has been implicated as a human pathogen.

### Identification of sarcocysts

When sequencing the 18S rRNA gene from *S. fayeri* sarcocysts isolated from horsemeat samples, it was difficult to obtain good sequences from all except a single Italian sample. In general, the 18S rRNA gene exhibits low variation, and it has therefore been widely used in the identification of parasites. However, after sequencing eight 18S rRNA sequences obtained from individual bradyzoites released from a single *S. fayeri* sarcocyst, the most divergent sequences among the eight samples exhibited 115 base substitutions, insertions, and deletions (out of 1,169 bp). The maximum and average genetic distances were 0.094 and 0.059, respectively. The genetic distances between *S. cruzi* (KT901167) and *S. hominis* (KF954731), *S. bovini* (KT901145), *S. bovifelis* (KC209742), and *S. hirsuta* (KT901163) were 0.050, 0.048, 0.045, and 0.058, respectively. Thus, the variation among bradyzoites from a single *S. fayeri* sarcocyst was higher than that among *Sarcocystis* spp. sarcocysts infecting cattle. Some *Sarcocystis* spp. have exhibited a high degree of intraspecific variation in the 18S rRNA gene, requiring the cloning of PCR products before sequencing [1,5,6]. It has been reported that this is probably due to multiple, slightly different variants of the gene in the genome, which may be caused by multiple copies of the 18S rRNA gene or by dissimilarities among gene copies from different merozoites within the sarcocyst [1]. Merozoites enter muscle cells and form metrocytes, which begin sarcocyst formation. The sarcocyst then grows through repeated asexual division of the merozoites [2,4]. However, all eight bradyzoite sequences indicated single chromatogram peaks, making it unlikely that the variation is due to the presence of many divergent 18S rRNA copies within the genome of each merozoite. Instead, the high sequence divergence among the DNA sequences from many bradyzoites may reflect differences in the genomes of different bradyzoites. Moreover, the fact that a single sequence was obtained from the Italian horsemeat sample suggests that high sequence variation may not occur in all *S. fayeri* sarcocysts. The mtDNA *cox1* sequences from the six cyst fragments of a single sarcocyst were 100% identical. Compared with these sequences, sequences from the four other sarcocysts isolated from the same Canadian horsemeat sample had 5–8 base substitutions between them. It is unlikely that this is due to the presence of other *Sarcocystis* spp. or other *S. fayeri* merozoites. Although each *S. fayeri* sarcocyst may develop from a single merozoite, the cause of such variation among 18S rRNA sequences of individual bradyzoites from a single *S. fayeri* sarcocyst is unknown. The phylogenetic analyses of the 18S rRNA and mtDNA *cox1* genes placed *S. fayeri* within clades that had canids and felids, respectively, as definitive hosts. The topologies of these phylogenetic trees were the same as those observed using different tree-building methods, such as the neighbor-joining method (data not shown). It is unknown why *S. fayeri* clusters with a clade of species with felid hosts, according to the phylogenetic analysis of the mtDNA *cox1* gene sequence, as the definitive hosts of *S. fayeri* are canids.

In contrast to sarcocysts isolated from horsemeat samples, *S. cruzi* and *S. hirsuta* sarcocysts isolated from beef samples did not exhibit high intraspecific variation in 18S rRNA sequences (data not shown). There was, however, intraspecific variation in *S. bovini* isolates from New Zealand beef samples, and it was necessary to employ cloning to obtain good sequences. In contrast, sequencing of the mtDNA *cox1* gene from *S. bovini* isolates resulted in a good sequence, similar to that of *S. fayeri*. Therefore, sequencing of the mtDNA *cox1* gene is more useful for identification of *Sarcocystis* species. This study also provides the first published partial sequence of the *S. fayeri* mtDNA *cox1* gene.
